# *Mycobacterium bovis* Infection in Red Foxes in Four Animal Tuberculosis Endemic Areas in France

**DOI:** 10.3390/microorganisms8071070

**Published:** 2020-07-17

**Authors:** Céline Richomme, Edouard Réveillaud, Jean-Louis Moyen, Perrine Sabatier, Krystel De Cruz, Lorraine Michelet, Maria Laura Boschiroli

**Affiliations:** 1 Nancy Laboratory for Rabies and Wildlife, ANSES, 54220 Malzéville, France; celine.richomme@anses.fr (C.R.); ps0103@laposte.net (P.S.); 2 Regional Directorate for Food, Agriculture and Forest of Nouvelle-Aquitaine, 87000 Limoges, France; edouard.reveillaud@agriculture.gouv.fr; 3 Laboratoire Départemental d’Analyse et de Recherche de la Dordogne, 24660 Coulounieix-Chamiers, France; jl.moyen@dordogne.fr; 4 Tuberculosis National Reference Laboratory, Laboratory for Animal Health, ANSES, University Paris-Est, 94700 Maisons-Alfort, France; krystel.decruz@anses.fr (K.D.C.); lorraine.michelet@anses.fr (L.M.)

**Keywords:** animal tuberculosis, red-foxes, multi-host communities, surveillance and control

## Abstract

In France, animal tuberculosis (TB) due to *Mycobacterium bovis* (*M.* *bovis*) affects a multi-host community that include cattle and wildlife species such as wild boars (*Sus scrofa*), badgers (*Meles meles*), or wild deer (*Cervus elaphus, Capreolus capreolus*). The involvement of foxes in the epidemiology of TB is fairly described in countries facing multispecies concerns. After the discovery of grouped cases of TB in foxes in a French TB endemic region, a study was implemented in the core of four TB endemic areas in Dordogne, Charente, Landes (departments of Nouvelle-Aquitaine region), and Côte-d’Or (Burgundy-Franche-Comté region). No infected fox was found in Côte-d’Or (n = 146), where in parallel TB in cattle and other wild species became sparse in the last years. In contrast, in Dordogne, Charente, and Landes, 13 (n = 184), 9 (n = 98) and 7 (n = 140) foxes were found infected by *M.* *bovis*, respectively, corresponding to 7.1% (CI_95%_ 3.8–11.8%), 9.2% (4.3–16.7%) and 5.0% (CI_95%_ 2.0–10.0%) prevalence rates, respectively. These infection rates are comparable with those observed in badgers and wild boar in these same three areas (ranging from 9 to 13.2% and 4.3 to 17.9%, respectively), where the number of cattle outbreaks has increased in the last 10-15 years. In each area, the genotypes of foxes’ *M.* *bovis* isolates were the same as those in local cattle and other wildlife species. None of the infected foxes presented TB-like gross lesions. *M.* *bovis* was found in the mesenteric lymph nodes of 28 foxes (68%). For the 12 foxes where retropharyngeal and respiratory lymph nodes were analyzed separately, *M.* *bovis* was present in the respiratory lymph nodes of eight individuals. With regard to excretion, appropriate samples were available for 12 infected foxes from Dordogne. *M.* *bovis* DNA was detected in the feces of five of these animals, four of which were infected in the mesenteric lymph nodes. Combined with the knowledge on the biology and ecology of foxes, the results of this study suggest that in areas where infection in cattle is still active in France, foxes might play a role of spillover host in the epidemiology of *M.* *bovis*.

## 1. Introduction

Animal tuberculosis (TB) due to *Mycobacterium bovis* is a zoonotic disease regarded in Europe not just as a livestock-problem only but as a concern for multi-host communities that include cattle and also wildlife species such as wild boars (*Sus scrofa*), red deer (*Cervus elaphus*), and badgers (*Meles meles*) [1]. It is recognized that the means to eradicate this disease is by controlling the domestic and wildlife reservoir(s), defined as epidemiologically connected populations in which the pathogen can be maintained and from which infection is transmitted to the target population [2]. Indeed, cattle-only TB measures are no longer efficient to prevent the increase of TB prevalence, which raised from 0.59% in 2010 to 0.86% in 2017 in Europe [3]. France is one of the officially tuberculosis free (OTF) EU member states facing, nonetheless, multi-host TB enzootic situations in several regions of the country [4]. If wildlife plays a significant role in disease persistence, a close knowledge on the composition of the infected host community is fundamental for any efficient future control intervention.

The red fox (*Vulpes vulpes*) had been considered as a spillover TB host with a minor role in TB epidemiology because only a sparse numbers of infected foxes had been found infected in highly prevalent TB regions such as in England [5]. In addition, the likelihood of *M. bovis* excretion had been considered low due to the absence of TB gross lesions in most of the cases, by analogy with what was observed in badgers [6]. However, in the last years high prevalence estimations of TB in foxes (up to 26.9%) were reported in multi-host TB endemic regions in the Iberian Peninsula [7]. In France, the number of foxes found infected since the early 2000s in TB endemic areas was usually low. However, in 2015, an outbreak was disclosed in this species [8]. Four out of 6 analyzed red foxes were found infected by *M. bovis* in a municipality of a TB-endemic area in Dordogne (Nouvelle-Aquitaine region, south-western France), where cattle, badgers, wild boar, and even roe deer and red deer are regularly found infected [4,8]. None of the four infected foxes exhibited TB gross lesions but all showed *M. bovis* fecal excretion and even potential urine and oropharyngeal mucus excretion for one of them [8].

In this context, a study was conducted in order to estimate the prevalence of infection in foxes in the core of enzootic areas of similar surfaces —about 500 km^2^— presenting common multi-host TB traits: one area in Dordogne, which include the fox formerly described outbreak, two other areas in Nouvelle-Aquitaine region, in Charente and Landes departments, and an area in Côte-d’Or department (Burgundy-Franche-Comté region, Eastern France). The study aimed also to collect data on exposure and excretion routes to elucidate the role of foxes in the epidemiology of TB in these areas as well to analyze the spatial link between infected foxes and local TB outbreaks in cattle.

## 2. Materials and Methods

### 2.1. Ethics Statement

All fox carcass used in the present study were provided by hunters who held the appropriate permits for hunting foxes under the supervision of local hunting federations, or trappers duly trained and authorized, supervised by pest control officers (historically called wolf-hunter, nowadays being state but volunteer officers in charge of pest control and who supervised trapping) in agreement with national regulations. As the study did not involve invasive procedures on live animals, no ethical approval was necessary.

### 2.2. Study Areas

**Figure 1:**
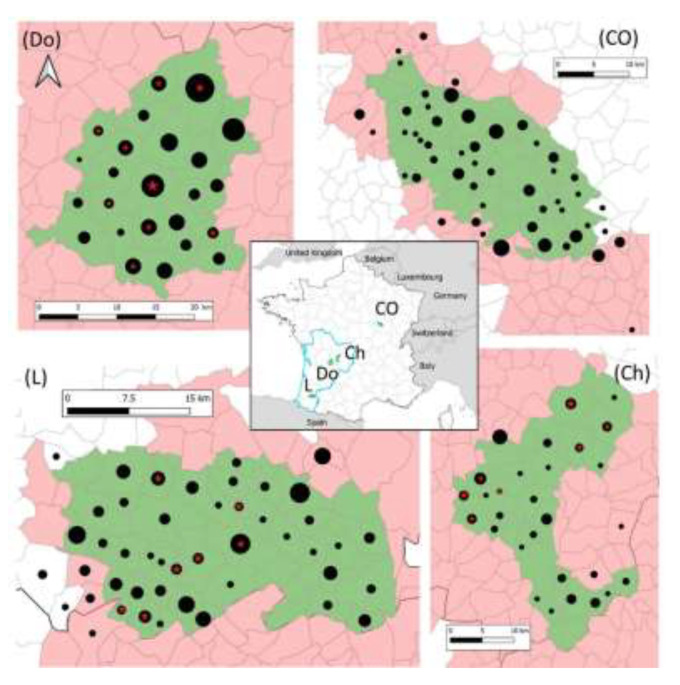
Location of sampled (black dots) and infected (red stars) foxes in the four studied areas (in green) in France. The grey lines delimit communes (administrative units). The communes in red form the infected areas where surveillance and management are implemented in cattle and in wildlife species (badgers, wild boar, and red deer). Black dots and red stars are positioned at the center of the communes and their size is proportional to the number of foxes (1 to 27 foxes for dots and 1 to 4 foxes for stars).

The study concerns four areas of about 500 km^2^ in the core of TB infected areas of Dordogne, Charente and Landes departments, in the South West of France (Nouvelle-Aquitaine region) and of Côte-d’Or department in Eastern France (Bourgogne-Franche-Comté region) (Figure 1 and Table 1). In these areas, *M. bovis* circulates in multi-host systems, TB is still prevalent in cattle and in several wild species [4] (Table 1).

### 2.3. Fox Sampling and Sample Collection

Sampling took place from March 2017 to January 2020. In Dordogne, foxes were collected in the whole study area until August 2018 (season #1) to estimate infection prevalence in the area. Then (season #2) animals from only two hotspots of the area were collected to maximize the chance of obtaining infected foxes and being able to better study exposure and, if possible, excretion routes. Two different protocols for sample collection during necropsies were employed in seasons 1 and 2 (see hereafter) (Table 2).

For estimating prevalence rates in Côte-d’Or, Charente, Landes, and Dordogne during season #1, an expected sample size of 130 foxes was determined to enable detection of *M. bovis* infection in foxes in each of the four areas, assuming an apparent prevalence of at least 3%, with a 95% confidence interval [9]. A sample size per municipality was calculated pro rata to its surface area; instructions to trappers and hunters working at a communal scale were provided accordingly.

For investigating natural exposure routes during season #2 in Dordogne, the goal was to study it on at least 10-12 infected foxes. Assuming a rough prevalence of around 10%, a sample size of 120 foxes was settled.

Data on sex (male, female), age class (juvenile: less than one year, adult: more than one year), municipality of trapping/hunting and date of trapping/hunting were recorded. In Dordogne, GPS coordinates of fox sampling/hunting location were also recorded.

Foxes were sent in double packaging to collect points and then to the local laboratories in charge of the necropsies. The following samples were collected at the lab: retropharyngeal lymph nodes (LN), respiratory LN (composed of tracheobronchial and mediastinal LN), mesenteric LN and any lesion suggestive of tuberculosis. In Dordogne, during season #1, feces, urine, or kidneys (if urine was absent), and oropharyngeal swabs were collected also.

### 2.4. Detection of M. bovis Infection

TB infection was determined by molecular diagnosis performed in (1) a pool of RP LN and resp. LN, and (2) in the mes. LN on animals from the four regions (Table 2), and in organs with gross lesions if they were observed. In Dordogne, during season #2, *M. bovis* DNA detection was performed separately (1) on the mes. LN, (2) the RP LN, and (3) the resp. LN. For foxes found infected in Dordogne during season #1, feces, urine, or kidneys (if urine was not available), and tracheal swabs were analyzed by molecular diagnosis. When positive, these samples were submitted to bacterial culture.

DNA extraction was performed after mechanical lysis using an LSI MagVetTM Universal Isolation Kit (Life Technologies) with a KingFisherTM Flex automate (Thermo Scientific), following the manufacturer’s instructions. For confirming *Mycobacterium tuberculosis* complex (MTBC) the LSI VetMAX^TM^ MTBC Real-Time PCR kit (Life Technologies), which targets IS*6110* and a PCR based on IS*1081* were employed [10]. IS*1561′* and Rv1510 (RD4) based PCRs were used to differentiate *M. microti* from *M. bovis* infections [10]. Spoligotyping by Luminex, as described by Zhang et al. [11], using TB-SPOL kits purchased from Beamedex® (Beamedex SAS, Orsay, France) on Bio-PLex 200/Luminex 200® was also employed on PCR positive samples for intra-MTBC differentiation and genotyping. The presence or absence of the 43 spacer sequences contained in the DR locus is represented in a binary code of 43 entries. Spoligotypes are named according to an agreed international convention (www.mbovis.org). Results were interpreted following the manufacturer’s recommendations and by comparison with negative and positive controls.

Bacterial culture was performed in parallel with PCR in foxes from Dordogne. In foxes collected in Charente, Landes, and Côte-d’Or, bacterial culture was performed only if molecular diagnosis was positive. Culture was done following the protocol established by the French NRL (NF U 47–104) for isolation of *M. bovis*. Two to 5 g of sampled tissues were crushed with a 4% sulfuric acid solution to decontaminate the tissue. After 10 min, the acid was neutralised by adding a 6% sodium hydroxide solution. After decontamination, the supernatant was seeded on two different media: Löwenstein-Jensen and Coletsos. All seeded media were incubated at 37 °C +/− 3 °C for three months and exanimated every 2 weeks. The isolated MTBC colonies were confirmed by DNA amplification [12] targeting the IS*6110* sequence present in all species of MTBC [13], and *M. bovis* was confirmed by spoligotyping as described above identified with the same molecular diagnosis methodology described in the previous section. Fecal samples were decontaminated with 4% NaOH (w/v) for 15 min at 37+/−2 °C and neutralized with 10% H2SO4 (v/v). All samples were inoculated onto Modified 7H11 medium (BD DifcoTM Mycobacteria 7H11 Agar, BD Biosciences, New Jersey, USA).

### 2.5. Data Analysis

An infected fox was defined as an animal with an analytical result demonstrating *M. bovis* infection by molecular diagnosis. Apparent prevalence in each of the 4 areas and 95% confidence intervals (CI_95%_) were calculated using exact binomial tests. For foxes from Nouvelle-Aquitaine region, a chisquare test (Chi^2^) was used to attest or not of a statically significant difference of the prevalence in each of the three areas, and in each gender.

For foxes collected in Dordogne during season #1 (2017–2018), GPS coordinates of fox sampling sites were available. We studied the spatial relationship between the distribution of infected foxes during season #1 and the plots grazed by cattle from a *M. bovis* infected herd two years before and two years after the sampling, i.e., from 2015 to 2019 (hereafter called « cattle outbreak pastures ») (Source of data: French ministry for Agriculture). In a first approach, we described the distance in meters between infected fox sampling site and the edge of the nearest cattle outbreak pasture. Then, we analyzed the proximity of fox sampling sites (for infected or non-infected foxes) to infected cattle pastures using a bootstrap method. The null hypothesis was that the infection status of foxes, infected or non-infected, was independent of their distance to infected cattle pastures, called D. We calculated the median value of D for infected and non-infected foxes for the observed dataset. Then, keeping the same number of infected and non-infected foxes, the status of foxes was randomly reassigned, to obtain a new sampling of the distance D under the null hypothesis. This procedure was repeated 10000 times, calculating for each simulation the mean value of D for the infected and non-infected individuals. Finally, the distribution of these median values enabled us to calculate the empirical p-value of the null hypothesis test as proportion of samples simulated under the null hypothesis for which the median value of D was lower than the observed value.

Statistical tests were considered significant if the *p*-value was < 0.05. Spatial and statistical analyses were performed using QGIS® software [14] and its extension NNjoin and R Studio® software [15].

## 3. Results

No infected fox was found in Côte-d’Or (n = 146). In Dordogne (season #1), Charente, and Landes, 13 (n = 184), 9 (n = 98), and 7 (n = 140) were found infected by *M. bovis*, with prevalence of 7.1%, 9.2%, and 5.0%, respectively (Table 3). In Nouvelle-Aquitaine, the prevalence in the three areas were not statistically different (Chi^2^, *p*-value = 0.4239). In these areas, prevalence in males (11.8% (CI_95%_: 7.6–17.2); n = 195) was higher than in females (5.5% (2.5–10.2); n = 163) but the difference was not statistically different (Chi^2^, *p*-value = 0.3801). The age class was registered for 372 individuals: one juvenile on 47 analyzed and 23 adults on 325 were infected.

During season #2 in Dordogne, 12 foxes were found infected among 95 foxes collected in the two hot spots where infected foxes had been found in season #1. Eight foxes exhibited infection in mesenteric LN, 8 in respiratory LN, and 3 in retropharyngeal LN (Table S1).

In each area, the genotype of the *M. bovis* strain that infected foxes was the same as those reported in cattle and other wildlife species in the same area: SB0120 in Dordogne and Charente, and SB0821 in Landes.

None of the infected foxes presented gross TB-like lesions.

When bacteriology and PCR were performed in parallel in Dordogne, culture positive samples were found among foxes that had PCR positives samples (two out of the 13 infected during season #1 and four out of the 12 infected during season #2) (Table S1).

Regarding *M. bovis* exposure, infection was disclosed in the mesenteric lymph nodes of 28 foxes (68%) among the 41 foxes found infected during the whole study, (Table 4 and Table S1), suggesting that infection took place by ingestion of infected food. For foxes with *M. bovis* presence in the pool of retropharyngeal LN and respiratory LN, it was not possible to determine if only one or both type of LNs were infected and therefore which type of exposure took place. When retropharyngeal and respiratory LN were analyzed separately (n=12), for one fox, *M. bovis* was found in the former NLs but not in the latter, which can also be the result of the oral infection during mastication of infected food (Table 4). *M. bovis* was present in the respiratory LN of eight foxes (Table 4 and Table S1) which could be the result of an infection by aerosols produced from an infected animal or material (dry soil in sett, but in pastures or cattle food in farms).

With regards to excretion, appropriate samples were available for 12 infected foxes from Dordogne. *M. bovis* DNA was detected in the feces of five of these animals, four of which were infected in the mesenteric lymph nodes (Table S1). These findings reveal a probable fecal excretion of *M. bovis*, even if live bacteria could not be confirmed by culture. Urine, or kidneys if urine was not available, and tracheal swabs were all negative by PCR.

Concerning spatial analysis, five of the 13 foxes found infected in 2017–2018 in Dordogne were trapped at fences of cattle pastures of farms found infected in 2016 or 2017. In addition, five foxes were collected within 300 m of cattle pastures of farms found infected between 2016 and 2019, and the remaining three infected foxes were collected approximately 1 km away (maximum = 1064 m) from any cattle outbreak. The median of the distance between the fox-sampling site and the center of the plots grazed by cattle from an infected farm between 2015 and 2019 is estimated to be of 301 m for infected foxes and 643 m for negative foxes. However, the result of the bootstrap analysis shows that there is no statistic demonstration of any spatial association between infection in foxes and cattle outbreak plots (*p* = 0.07 > 0.05).

## 4. Discussion

Our study demonstrates that in infected areas where TB is still active being transmitted in cattle and wildlife, foxes are involved in TB epidemiology and paves the way for future projects for studying its role in multi-host systems in Europe. Indeed, previous studies describing TB infection in foxes are scarce and reports on epidemiological data in wild red fox populations concern only England and the Iberian Peninsula [5,7,16,17,18,19]. Here, in the three studied TB endemic areas of South west of France, where the number of cattle outbreaks has increased during the last years 10–15 years, infection rates in foxes are of the same order of magnitude than those observed in badgers and in wild boars in these same three areas (ranging from 9 to 13.2% and 4.3 to 17.9%, respectively (Table 1). Before the present study, four foxes were found infected among 102 foxes analyzed between 2005 and 2014 in Côte-d’Or (unpublished data, source: French ministry for Agriculture). During that period, the number of cattle outbreaks in this region was high. In our study, no fox was found infected (n = 146) whereas in parallel TB in cattle has decreased (only two outbreaks in 2019; source: French ministry for Agriculture) and infection in other wild species has also decreased [4]. Therefore, it appears that in France, when cattle, considered as the main *M. bovis* reservoir [20] is barely affected by TB, infection in foxes is either inexistent or at a too low prevalence rate to be detected. However, when TB is still endemic in extensive cattle breeding areas, foxes take part of the wild TB community. 

Infected foxes were trapped closer to spots grazed by cattle from an infected herd (median of distance of 301 m for infected foxes and 643 m for non-infected foxes). However, the spatial analyses we carried out—employed before in one of our previous studies to test spatial association between positive wild boar in serology and cattle outbreaks [21]—did not elucidate any statically significant link between pastures of infected herd and trapping sites of infected foxes. Pastures are probably not the only sites of contact between these two animal species explaining fox/cattle exposure. In France, TB is endemic in extensive beef-cattle breeding regions. Cattle stay during long periods in pastures which are frequented by foxes to chase rodents [22] or eat earthworms [23]—predation intensity depending on weather conditions and distribution of preys—or to consume the placenta or post-calving discharges of cows. In France, a study carried out in Côte d’Or based on camera monitoring at 101 points located in pastures and farm buildings and considered attractive for wildlife (water and food access), revealed that the fox is the most frequent wild species to visit these sites compared to badgers and wild ungulates [24,25]. Previous study in England and Ireland also showed that foxes visited cattle buildings very frequently [26,27]. In cattle sheds, foxes were observed predating rodents [26] but also in feeding troughs [25]. This behaviour can lead to indirect exposure to *M. bovis* bacilli excreted by cattle. The interface between cattle and foxes is most probably indirect as foxes are less often seen in direct contact with cattle than for instance with badgers [5,28]. Interspecific contacts can also occur between foxes and wild ungulates, especially on baited places and waterholes [29], and between foxes and badgers. Given that GPS location of badgers, wild boar or deer was not available, spatial location between them, and foxes was not possible in our study. However, data on these last interactions have been obtained in the framework of studies carried out in England and in France where burrows were baited with the candidate bait for vaccination of badgers against tuberculosis. While in England very few foxes were observed coming to burrows (0.4% of all observations) [30], in France, it was the most frequent wild species to visit setts [31]. Some observations (exit or entry into the sett) suggested that foxes occupy burrows left temporarily vacant by badgers.

The higher frequency of digestive infection found in our study reveals that foxes are often exposed by the oral route. This result is in accordance with Portuguese data collected in an area where TB is highly prevalent in Suidae and Cervidae; Matos et al. suggested that the digestive infection they observed could be explained by wild ungulates carcass consumption [7]. Even though hunted-harvested carcass is compulsory in French infected areas, it cannot be ruled out that some of them are not left behind or found by hunters. Besides, infection by cattle contaminated food cannot be excluded. The respiratory infection was also confirmed in our study. Contaminated droplets or dust particle aerosols could be at the origin of these infections. Infected soil particles could be present in contaminated badger sets shared with foxes.

In England, foxes TB prevalence was significantly higher in females than males [5], but for the authors it was not clear whether this was because of a difference in susceptibility or enhanced disease-induced mortality among males. In the present study, males appear to be twice as infected than females, albeit no statistically significant differences between sexes were determined. A more robust data collection would be necessary to confirm this trend. Regardless of the sex, no animal exhibited TB gross lesions. Similarly, no severe pathology was observed in any fox collected in England (n = 993 [18] or n = 27 [5]) or in Portugal where *M. bovis* infection was detected by microbiological culture and PCR in organs without any visible lesion (n = 52). This absence of macroscopic lesion could reveal the ability of the fox to control TB infection.

Transmission of *M. bovis* via droplet aerosols produced by the respiratory tract of infected foxes cannot to be ruled out even in the absence of if visible lesions. Indeed, badgers with no visible lesions can excrete by the respiratory way [32]. Immuno-histochemical and other histological analyses would be useful in future studies to assess the presence of microscopic lesions of foxes infected tissues for better understating *M. bovis* infection in this species. With regards transmission to cattle, the most striking result observed in the present study is the detection of *M. bovis* DNA in feces of some infected foxes, many of them being infected in the mesenteric lymph nodes, suggesting faecal excretion of *M. bovis,* as observed in badgers [33]. As mentioned beforehand, foxes visit frequently cattle sheds to feed and they were also observed defecating and urinating in cattle feeding troughs [25]. Ongoing *M. bovis* whole genome sequence studies (including cattle and fox strains) will help us to elucidate if transmission from foxes to cattle occur and which is the infectious relationship between these hosts in the different regions of our study.

## 5. Conclusions

The results of the present study strongly suggest that in areas where infection in cattle is still active in a multi-host transmission system, foxes can be involved in the epidemiology of *M. bovis*. In certain conditions still to be elucidated, foxes get infected when they are exposed to *M. bovis*, they do not develop a severe pathology, but they could be capable of excreting bacteria via feces. Given that these species get in close contact to cattle, *M. bovis* spill-back transmission between the two species seems possible. This raises challenging questions in terms of TB control because the presence of infected foxes could hamper local eradication plans. Any measure to address the risk of transmission from foxes clearly needs to consider the biology and behavior of the fox in response to them.

## Figures and Tables

**Table 1:** Characteristics of the four study areas. Number of cattle outbreaks, mean prevalence in badgers and wild boar and cases in wild deer (roe deer and red deer) are estimated based on national surveillance data from 2015 to 2018 (Source: French ministry of Agriculture).

Region	Nouvelle-Aquitaine			Burgundy – Franche-Comté
Department	Dordogne (Do)	Charente (Ch)	Landes (L)	Côte-d’Or (CO)
Surface of the area (in km^2^)	525	539	504	484
Number of cattle outbreaks	35	13	31	22
Mean prevalence in badger (# infected/# analyzed)	11.1% (15/135)	9.0% (29/321)	13.2% (20/152)	6.2% (27/432)
Mean prevalence in wild boar (# infected/# analyzed)	4.3% (8/185)	7.8% (20/255)	17.9% (7/39)	2.2% (6/269)
Number of cases in wild deer	2	1	0	2

**Table 2:** Summary of the material and methods used in the four study areas.

Region	Department		Sampling Collection	Tissues Used for *M. bovis* Detection	Data Analysis			
					Apparent Prevalence	Exposure Routes	Excretion Routes	Fox Infection/Cattle Outbreak Pastures Spatial Relationship
Burgundy – Franche-Comté	Côte-d’Or		June 2018 – November 2019	Pool (RP LN + resp. LN), mes. LN	x	x	-	-
Nouvelle-Aquitaine	Charente				x	x	-	-
	Landes				x	x		-
	Dordogne	Season #1	March 2017 to August 2018	Pool (RP LN + resp. LN), mes. LN, feces ^1^, urine ^1,2^, oropharyngeal swab ^1^	x	x	x	x
		Season #2	August 2018 to November 2019	Separately RP LN, resp. LN, mes. LN	-	x	-	-

^1^ for foxes found positive on LN. ^2^ or kidneys if urine absent. x: done / -: not done.

**Table 3:** *M. bovis* infection of foxes in three infected areas of Nouvelle-Aquitaine and in the core infected area of Côte-d’Or, France.

	Number of foxes		Prevalence (%)	95% Confidence Interval
	Analyzed	Infected		
Charente	98	9	9.2	4.3–16.7
Dordogne (season #1)	184	13	7.1	3.8–11.8
Landes	140	7	5.0	2.0–10.0
Nouvelle-Aquitaine *	422	29	6.9	4.6–9.7
Côte-d’Or	146	0	-	0–2.5

* Prevalence in the 3 infected areas of Nouvelle-Aquitaine were not statistically different (Chi^2^, *p*-value = 0.4239).

**Table 4:** Results of *M. bovis* detection in the different tissues of the infected foxes found in Nouvelle-Aquitaine. LN: lymph nodes; RP: retropharyngeal; resp: respiratory.

	*M. bovis* Detection		
Tissues Analyzed	Total Analyzed	Culture Positive	PCR Positive
LN mesenteric	41	4	28
LN RP + resp pooled	29	2	26
LN RP separately	12	3	1
LN resp separately	12	1	8
Feces	12	0	5 ^(1)^

^(1)^ For 4 foxes, *M. bovis* was detected in the mesenteric LN.

## Supplementary Materials

The following are available online at https://www.mdpi.com/2076-2607/8/7/1070/s1, Table S1: Results of *M. bovis* detection by molecular diagnosis in tissues and feces, when available (NA when not), for each of the 41 foxes found infected in Nouvelle-Aquitaine.
