# Supplementary material for: Mycobacterium bovis Infection in Red Foxes in Four Animal Tuberculosis Endemic Areas in France
Source: Microorganisms. 2020 Jul 17;8(7):1070. doi: 10.3390/microorganisms8071070 (PMC7409206; doi:10.3390/microorganisms8071070)
Supplement: Supplementary file 1 [file microorganisms-08-01070-s001.pdf]

Table S1. Results of *M. bovis* detection by molecular diagnosis in tissues and feces, when available (NA when not), for each of the 41 foxes found infected in Nouvelle-Aquitaine. LN: lymph nodes; RP: retropharyngeal; resp: respiratory. NR: not reported. In bold font, tissues which were positive in bacterial culture. In underlined font feces of foxes in which *M. bovis* was also detected in mesenteric LN.

ID fox	Dept	Season	Date of capture	Sex	Age	NL mes	NL RP + resp	NL RP	NL resp	Feces
181210017630	Charente	-	08/12/2018	Male	Adult	NEG	POS	-	-	NA
181211017706	Charente	-	09/12/2018	Male	Adult	POS	NEG	-	-	NA
190102000053	Charente	-	30/12/2018	Female	Adult	NEG	POS	-	-	NA
190115000848	Charente	-	13/01/2019	Female	Adult	NEG	POS	-	-	NA
190226003554	Charente	-	18/02/2019	Male	Adult	POS	NEG	-	-	NA
190328005539	Charente	-	28/03/2019	Male	Adult	NEG	POS	-	-	NA
191105017951	Charente	-	05/11/2019	Female	Adult	POS	POS	-	-	NA
191112018158	Charente	-	12/11/2019	Female	Adult	POS	POS	-	-	NA
200121001108	Charente	-	18/01/2020	Female	Adult	POS	NEG	-	-	NA
170425020397	Dordogne	#1	31/03/2017	Female	Adult	POS	POS	-	-	NEG
170517023926	Dordogne	#1	27/04/2017	Female	Adult	POS	**POS**	-	-	<u>POS</u>
170522024668	Dordogne	#1	29/04/2017	Female	Juvenile	POS	POS	-	-	NEG
170809036689	Dordogne	#1	28/07/2017	Male	Adult	NEG	POS	-	-	POS
170809036690	Dordogne	#1	28/07/2017	NR	Adult	POS	POS	-	-	<u>POS</u>
170809036691	Dordogne	#1	28/07/2017	NR	NR	POS	**POS**	-	-	<u>POS</u>
170809036694	Dordogne	#1	01/08/2017	Female	Adult	NEG	POS	-	-	NEG
170808036524	Dordogne	#1	03/08/2017	Female	Adult	NEG	POS	-	-	NEG
170822038207	Dordogne	#1	04/08/2017	Female	Adult	POS	POS	-	-	NEG
170816037526	Dordogne	#1	04/08/2017	Female	Adult	POS	POS	-	-	<u>POS</u>
170823038426	Dordogne	#1	11/08/2017	Male	Adult	NEG	POS	-	-	NEG
180524023861	Dordogne	#1	16/05/2018	Male	Adult	POS	POS	-	-	NA
180525024451	Dordogne	#2	20/05/2018	Male	NR	NEG	-	NEG	POS	NA
180525024452	Dordogne	#2	24/05/2018	Male	Juvenile	NEG	-	NEG	POS	NA
180814038263	Dordogne	#1	26/06/2018	Female	Adult	POS	-	NEG	POS	NA
180806036886	Dordogne	#2	28/07/2018	Female	Adult	POS	-	**NEG**	POS	NA
180814038265	Dordogne	#2	31/07/2018	NR	Juvenile	POS	-	NEG	NEG	NA
180822039364	Dordogne	#2	01/08/2018	Male	Adult	POS	-	NEG	NEG	NA
180814038270	Dordogne	#2	07/08/2018	Male	Adult	POS	-	NEG	POS	NA
180814038267	Dordogne	#2	07/08/2018	Female	Juvenile	POS	-	**POS**	POS	NA
180814038272	Dordogne	#2	07/08/2018	Male	Juvenile	NEG	-	NEG	POS	NA
180814038259	Dordogne	#2	08/08/2018	NR	NR	NEG	-	POS	NEG	NA
180829040338	Dordogne	#2	19/08/2018	Female	Adult	**POS**	-	**NEG**	**POS**	NA
180822039375	Dordogne	#2	10/10/2018	NR	NR	**POS**	-	NEG	NEG	NA
190430018822	Dordogne	#2	16/03/2019	NR	NR	NEG	POS	-	-	NEG
2589332	Landes	-	23/11/2018	Female	NR	**POS**	POS	-	-	NA
2589331	Landes	-	13/12/2018	Male	NR	POS	POS	-	-	NA
2589528	Landes	-	06/01/2019	Male	NR	POS	POS	-	-	NA
238802	Landes	-	18/01/2019	Male	Adult	POS	POS	-	-	NA
2589341	Landes	-	23/01/2019	Male	Adult	POS	POS	-	-	NA
2589568	Landes	-	21/03/2019	Male	NR	**POS**	POS	-	-	NA
238128	Landes	-	30/03/2019	Male	Adult	POS	POS	-	-	NA
